# Phospholipid–Prednisolone Conjugates for Targeted IBD Therapy: Structural Design, Synthetic Approaches, Optimization, and Computational Evaluation

**DOI:** 10.3390/molecules31173057

**Published:** 2026-08-31

**Authors:** Sapir Ifrah, Adi Jabarin, Ludmila Yarmolinsky, Keren Cohen-Hagai, Mordechai Deutsch, Arik Dahan, Shimon Ben-Shabat

**Affiliations:** 1 Department of Clinical Pharmacology, School of Pharmacy, Faculty of Health Sciences, Ben-Gurion University of the Negev, Beer-Sheva 8410501, Israel; sapirgar@post.bgu.ac.il (S.I.); adi.jbaren@gmail.com (A.J.); yludmila@post.bgu.ac.il (L.Y.); 2 Department of Nephrology and Hypertension, Meir Medical Center, Kfar Saba 4428164, Israelmotti.jsc@gmail.com (M.D.)

**Keywords:** drug targeting, IBD, PLA_2_, phospholipid, prednisolone, prodrug approach

## Abstract

Inflammatory bowel disease (IBD), including Crohn’s disease and ulcerative colitis, is a chronic disorder characterized by persistent intestinal inflammation. Although corticosteroids such as prednisolone effectively control disease symptoms, prolonged treatment is associated with severe side effects, including immune suppression and metabolic disturbances. To enable site-specific drug delivery, four phospholipid–linker–prednisolone conjugates were designed and synthesized as prodrugs targeting the overexpression of phospholipase A_2_ (PLA_2_) in inflamed intestinal tissues. The conjugates were prepared using a reversed synthetic strategy, in which the phospholipid–linker scaffold was assembled before drug coupling. The effect of spacer length on molecular conformation and predicted structural determinants of enzymatic activation was investigated through in silico analysis. Molecular docking simulations performed using the AutoDock Vina v1.2.7 framework, followed by structural and distance analysis in UCSF Chimera and 50 ns molecular dynamics simulations in GROMACS, suggested that linker length may influence ligand orientation, conformational orientation, ligand stability, and the spatial positioning of the ester bond relative to the catalytic histidine residue within the PLA_2_ active site. Among the two conjugates examined in detail by molecular dynamics, C6 maintained comparatively lower ligand mobility and a shorter average distance to the catalytic residue His47 than C12. These computational findings provide structural insights that may guide the future design and optimization of phospholipid-based corticosteroid prodrugs for targeted IBD therapy.

## 1. Introduction

Inflammatory bowel diseases (IBD), including Crohn’s disease and ulcerative colitis, are chronic relapsing disorders characterized by persistent inflammation of the gastrointestinal tract [1]. The disease is associated with disruption of the intestinal epithelial barrier, dysregulated immune responses, and progressive mucosal damage, all of which significantly impair patients’ quality of life. IBD pathogenesis involves a complex interplay between genetic susceptibility, environmental triggers, intestinal dysbiosis, and aberrant mucosal immune activation, leading to excessive secretion of pro-inflammatory cytokines and recurrent cycles of remission and relapse [2,3]. Recent studies further highlight the complex interactions between cellular stress, inflammatory processes, and microbiota-related mechanisms, emphasizing the multifactorial nature of inflammatory conditions and the need for more selective therapeutic strategies [4,5].

IBD represents a growing global health burden, with continuously increasing prevalence worldwide [6]. Because the disease often begins in early adulthood, patients frequently require long-term treatment for decades, creating substantial clinical, social, and economic challenges [7]. This long disease duration highlights the urgent need for therapeutic strategies that combine high anti-inflammatory efficacy with improved long-term safety.

Corticosteroids remain among the most effective agents for controlling acute intestinal inflammation, primarily by suppressing pro-inflammatory cytokines such as IL-1β, TNF-α, IL-6, IL-8, and GM-CSF [8,9]. Prednisone is metabolically converted in the liver to prednisolone, its active form [10]. Prednisolone exerts its therapeutic activity through glucocorticoid receptor-mediated transcriptional regulation, resulting in inhibition of leukocyte recruitment, suppression of inflammatory mediator synthesis, and reduction in mucosal edema [11]. However, prolonged corticosteroid therapy is associated with serious adverse effects, including immunosuppression, osteoporosis, and metabolic disturbances [12].

To overcome these limitations, targeted drug delivery strategies have attracted considerable attention [13,14,15]. Current approaches for IBD therapy often rely on pH-dependent, time-dependent, or microflora-dependent colonic delivery systems [16,17,18]. However, these approaches may be limited by interpatient variability and by the fact that inflammation can occur at multiple sites throughout the gastrointestinal tract rather than exclusively in the colon [19,20].

**Scheme 1:**
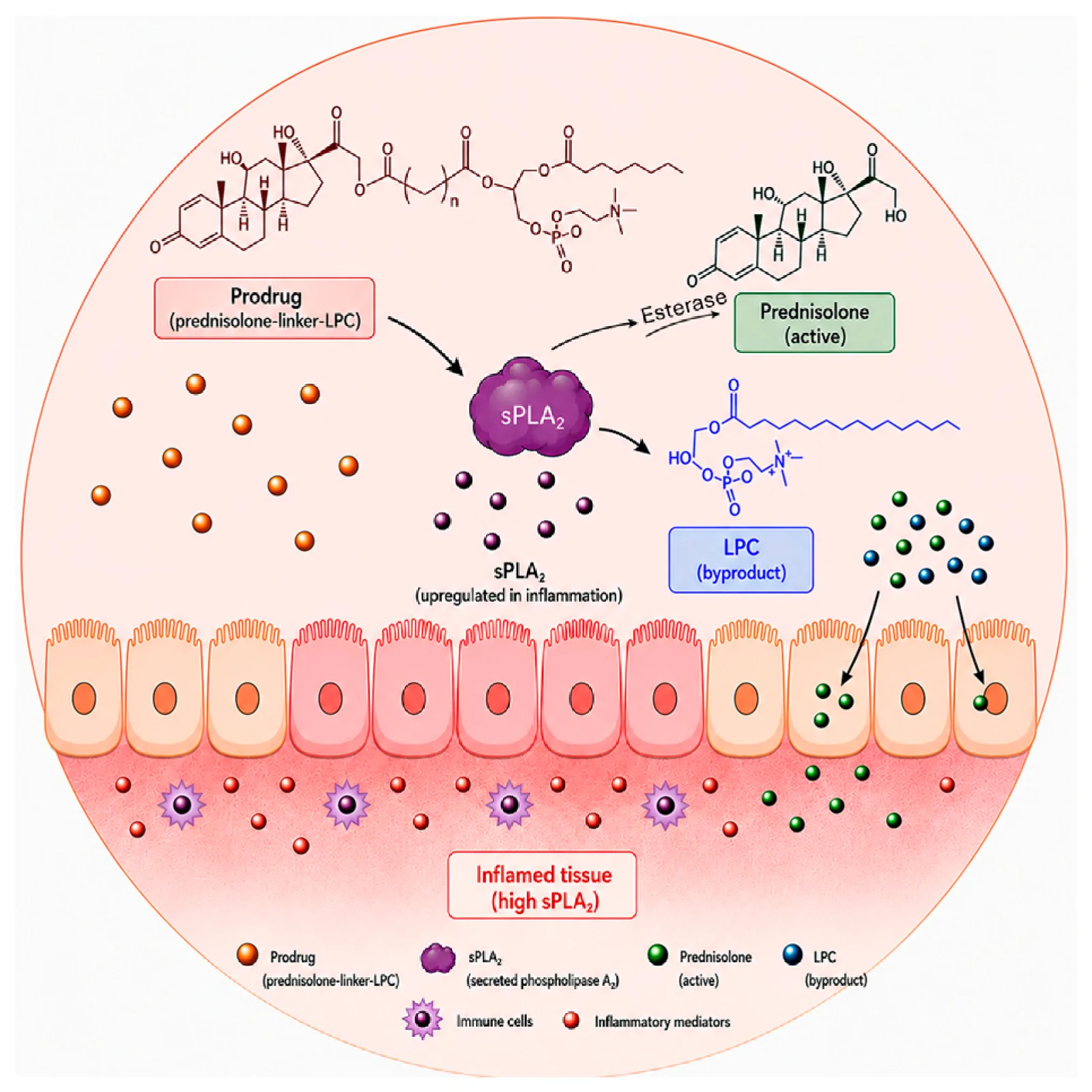
Schematic illustration of the proposed mechanism for targeted activation of the LPC–prednisolone prodrug in inflamed intestinal tissue: elevated extracellular sPLA_2_ secreted from inflamed epithelial and immune cells hydrolyzes the phospholipid prodrug at the site of inflammation, releasing active prednisolone locally to reduce inflammation and promote mucosal healing.

One promising alternative is the use of enzyme-responsive prodrugs based on phospholipase A_2_ (PLA_2_). Under physiological conditions, PLA_2_ expression remains relatively low, whereas in inflamed intestinal tissues its expression and activity are markedly elevated [21,22]. This enzyme specifically recognizes and hydrolyzes the sn-2 ester bond of phospholipids, releasing the corresponding lysophospholipid and fatty acid products [23]. Because the catalytic mechanism centers on a conserved histidine-containing active site, conjugation of therapeutic agents such as prednisolone to the sn-2 position provides a rational strategy for site-specific activation in inflamed tissues (Scheme 1) [24].

Our group has previously applied this concept to phospholipid-based prodrugs of diclofenac and cyclosporine [1,2]. These studies suggested that linker length plays an important role in determining the efficiency of PLA_2_-mediated activation and the dynamic positioning of the prodrug within the enzyme active site. In particular, previous computational and experimental studies have indicated that different drugs may favor different linker lengths to achieve a favorable catalytic orientation within the PLA_2_ active site.

In the present study, we adopted a reversed synthetic strategy, in which the phospholipid–linker scaffold was first assembled, followed by subsequent coupling to prednisolone. This reverse synthetic strategy represents a methodological advancement over our previous synthetic approach. In the present study, it enabled the successful synthesis of phospholipid–prednisolone conjugates. Because the phospholipid–linker intermediate can, in principle, be coupled with other therapeutic agents, this methodology may provide a useful framework for future development of additional phospholipid-based prodrugs.

In addition to synthetic development, computational modeling provides an important tool for rational prodrug design. Molecular docking and molecular dynamics (MD) simulations provide structural insights into linker-dependent conformational behavior, ligand orientation, conformational stability, and the spatial positioning of the ester bond relative to the catalytic histidine residue within the PLA_2_ active site [2,3]. Such in silico approaches significantly reduce experimental trial-and-error and may facilitate the selection of promising linker lengths for further experimental evaluation [24]. Molecular docking has become a widely applied computational technique in modern drug discovery, enabling the prediction of ligand–target binding modes, identification of key molecular interactions, and prioritization of candidate compounds before experimental testing [25,26]. By providing structural insights into ligand recognition and binding orientation, docking serves as an efficient screening tool that can guide rational molecular design while reducing the need for extensive experimental screening [25,26]. Although docking does not fully account for protein flexibility and dynamic solvent effects, it remains a valuable approach for early-stage evaluation and optimization of drug candidates [25]. To complement the docking analysis, MD simulations enable the assessment of complex stability, residue flexibility, and ligand orientation over time under explicit solvent conditions [27]. Within the limitations of the simulations performed, such simulations provide additional insights into the dynamic organization of enzyme–prodrug complexes and the accessibility of catalytically relevant conformations.

In the context of phospholipid-based prodrugs, molecular docking is particularly useful for assessing how linker length influences the positioning of the conjugate within the enzyme active site. Since the efficiency of PLA_2_-mediated activation depends not only on productive enzyme binding but also on the orientation of the cleavable ester bond relative to catalytic residues, docking can provide important preliminary information regarding the likelihood of productive enzyme–substrate interactions. Furthermore, MD simulations can provide insights into whether the predicted binding modes remain stable over time and whether linker length affects the dynamic accessibility of the catalytic region [24]. In this context, molecular docking and molecular dynamics simulations were employed in the present study as a structure-based screening tool to compare linker-dependent differences in predicted binding behavior and catalytic accessibility before biological evaluation.

Accordingly, the present study aimed to synthesize a series of phospholipid–linker–prednisolone conjugates with varying spacer lengths and to evaluate their conformational behavior and predicted structural determinants of PLA_2_-mediated activation using molecular docking, molecular dynamics simulations, and structural analysis.

## 2. Results

### 2.1. Chemistry

**Scheme 2:**
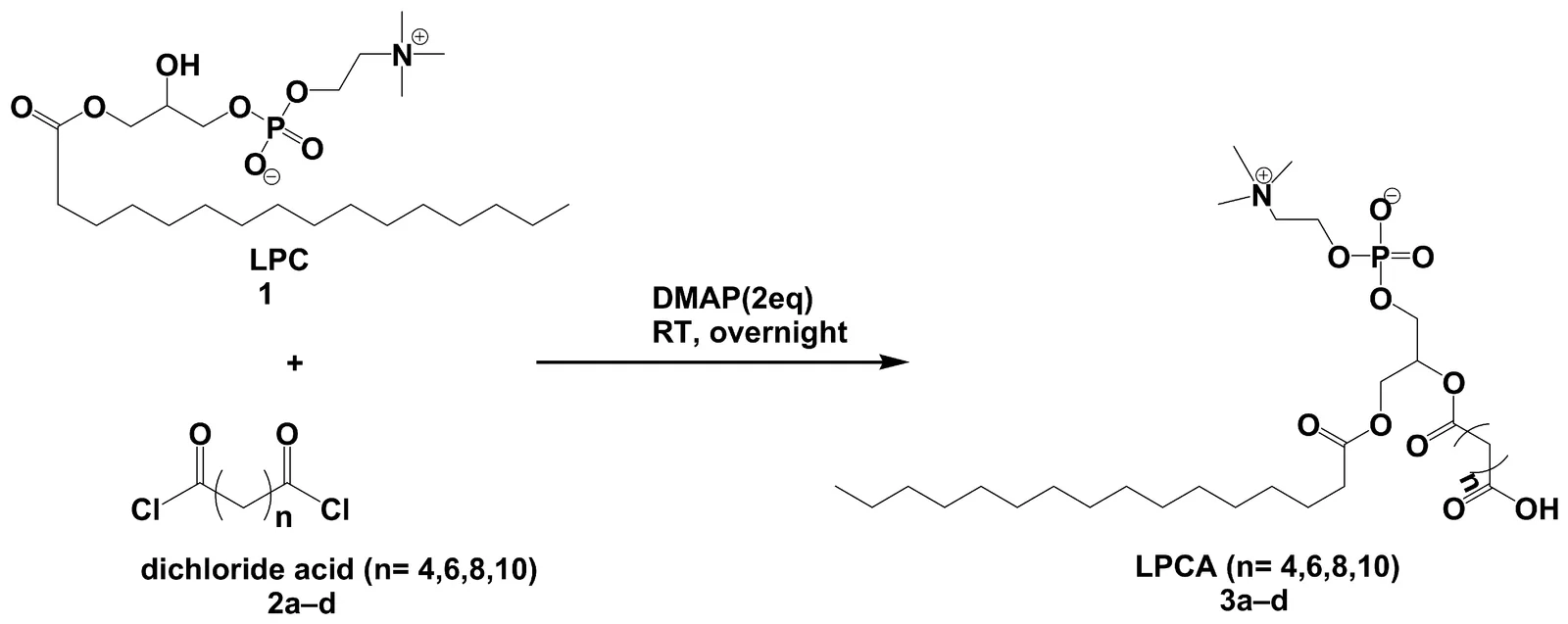
Synthesis of LPC–linker intermediates (**3a**–**d**).

The lysophosphatidylcholine (LPC)–linker intermediates, **3a**–**d**, were synthesized according to the procedure outlined in Scheme 2. Briefly, commercially available LPC 1 was reacted with the corresponding aliphatic diacid chloride derivatives **2a**–**d** in dichloromethane at room temperature in the presence of 4-dimethylaminopyridine (DMAP) as a catalytic base. The reactions were allowed to proceed overnight and monitored by thin-layer chromatography (TLC). Following completion, the crude reaction mixtures were purified by cartridge-based methanol/water extraction to afford the desired intermediates **3a**–**d**. During purification, the remaining acid chloride functionality was hydrolyzed to the corresponding carboxylic acid, yielding the phospholipid–linker intermediates used in the subsequent coupling step. LC-MS characterization of the isolated intermediates confirmed the expected molecular masses of the desired mono-substituted phospholipid–linker products, while no detectable signals corresponding to LPC–linker–LPC dimeric species were observed. In addition, the NMR data obtained for the purified intermediates and final conjugates were consistent with the expected structures, further supporting the selective formation of the desired products.

**Scheme 3:**
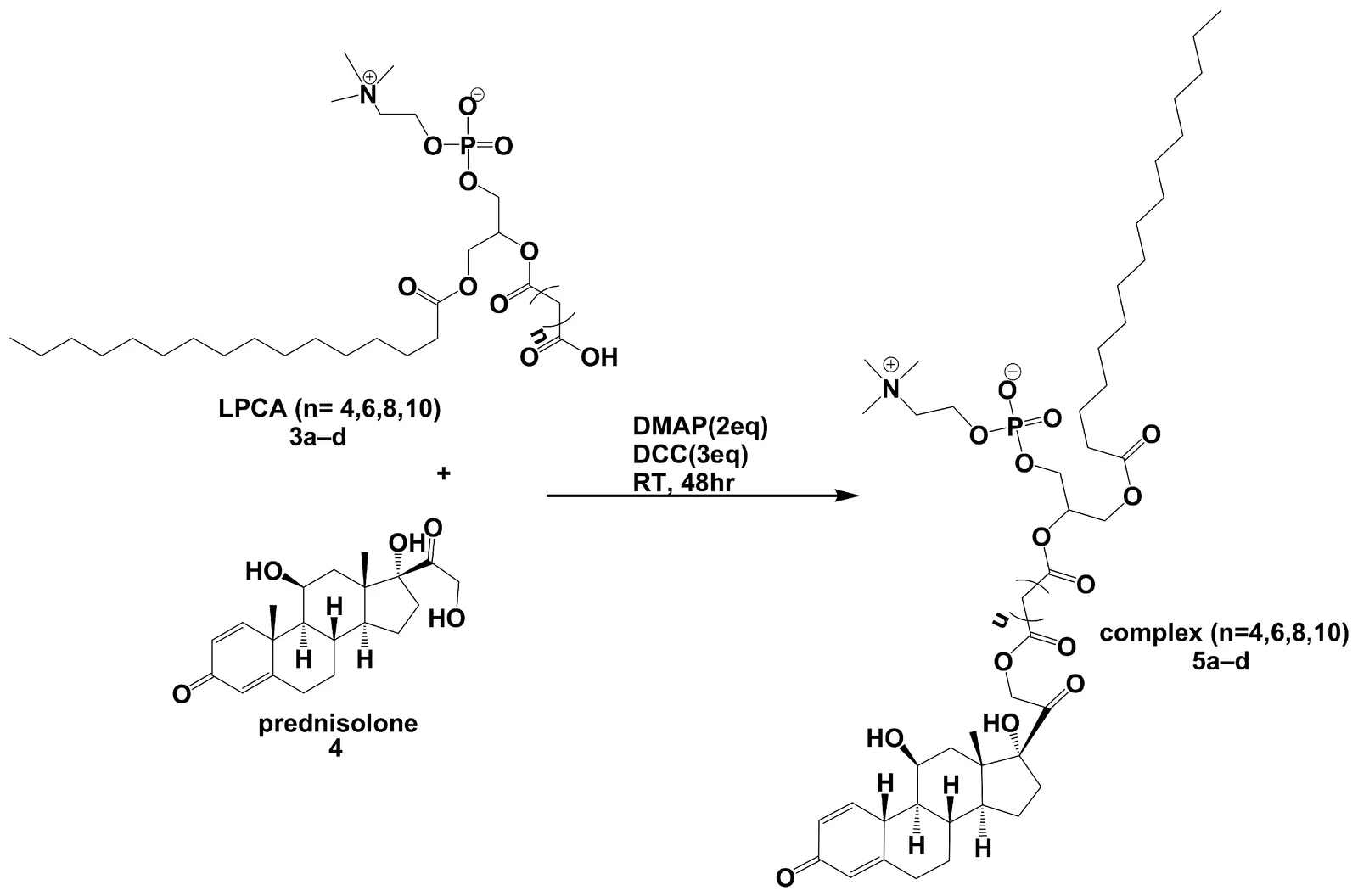
Synthesis of LPC–prednisolone drug conjugates with differences in linker lengths.

The final phospholipid–linker–prednisolone conjugates were subsequently obtained by coupling intermediates LPCA-C4 (**3a**), LPCA-C6 (**3b**), LPCA-C8 (**3c**), and LPCA-C10 (**3d**) with prednisolone **4**, as shown in Scheme 3. For this purpose, **3a**–**d** were reacted with prednisolone in distilled dichloromethane in the presence of N,N′-dicyclohexylcarbodiimide (DCC) and DMAP at room temperature for 48 h. After the reaction was completed, the solvent was evaporated under reduced pressure, and the crude products were dissolved in methanol. They were then purified by preparative HPLC using a gradient of IPA:MeOH:water to yield the desired phospholipid–linker–prednisolone conjugates. The synthesized intermediates were designated LPCA-C4 (**3a**), LPCA-C6 (**3b**), LPCA-C8 (**3c**), and LPCA-C10 (**3d**), while the final conjugates were designated LPC-C4-PR (**5a**), LPC-C6-PR (**5b**), LPC-C8-PR (**5c**), and LPC-C10-PR (**5d**).

### 2.2. Molecular Modeling Studies

#### 2.2.1. Molecular Docking Simulations

An initial molecular docking screen was performed to evaluate the interaction of LPC–prednisolone prodrugs bearing different linker lengths (C2–C12) with human secretory phospholipase A_2_ (sPLA_2_). Docking was conducted using AutoDock Vina v1.2.7 through the PyRx interface under hydrated and dehydrated receptor conditions, using an exhaustiveness value of 8. Multiple binding poses were generated for each conjugate and ranked according to their predicted binding affinity. The top-ranked pose of each conjugate was used for the initial comparison of linker-dependent binding behavior.

**Figure 1:**
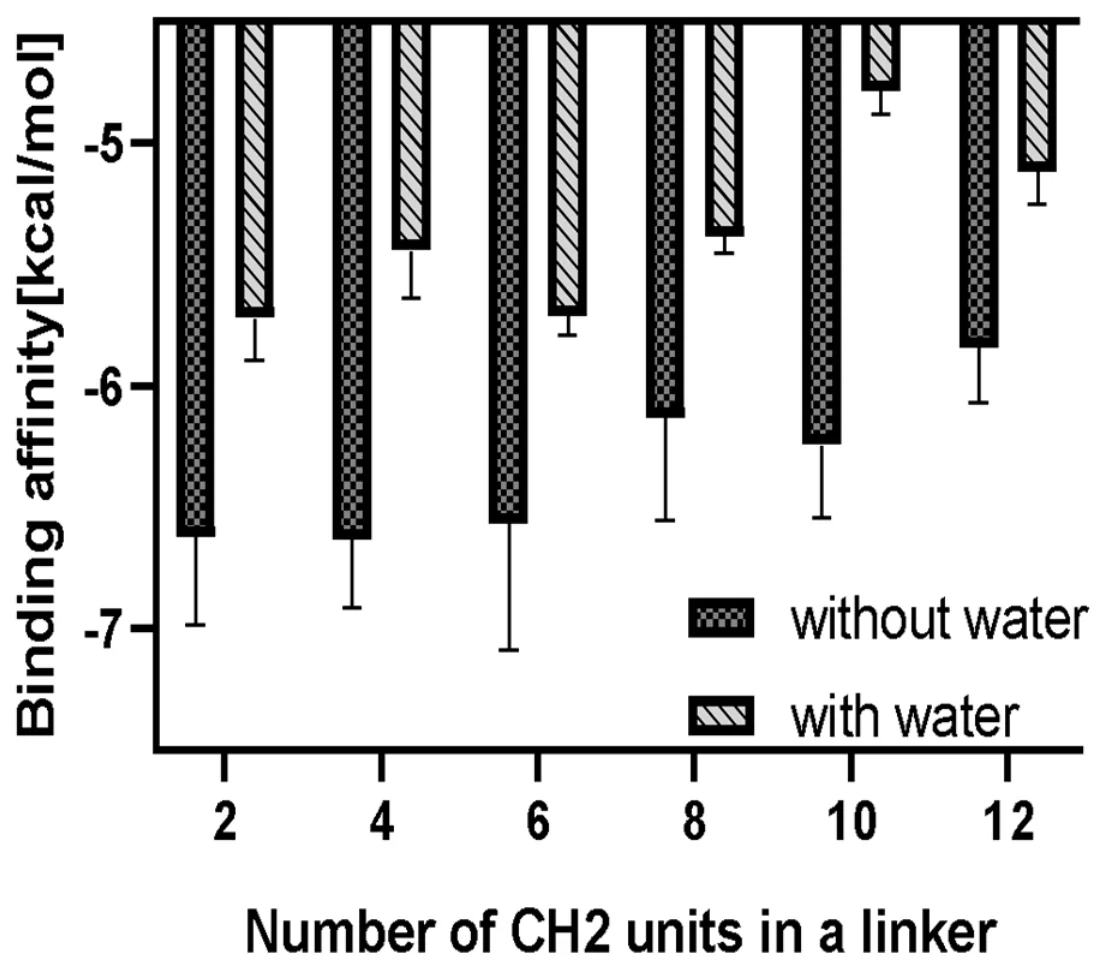
Initial docking screening of PL–prednisolone conjugates with varying linker lengths (C2–C12) toward human secretory phospholipase A_2_ under hydrated and dehydrated active-site conditions. Predicted binding affinities were obtained using AutoDock Vina v1.2.7 with an exhaustiveness value of 8.

Importantly, comparison between docking conditions suggests that the presence of solvent influences the absolute binding energies while preserving the overall trend. As illustrated in Figure 1, inclusion of water-related effects leads to moderate shifts in binding affinity values, reflecting solvent-mediated stabilization and hydrophobic interactions. However, the relative ranking of linker lengths remains consistent, with intermediate spacers (C4–C6) maintaining superior binding characteristics.

These findings suggest a tendency for intermediate linker lengths to adopt more favorable binding modes within the catalytic pocket of human sPLA_2_. Shorter and intermediate spacers appeared to favor more effective positioning of the prednisolone moiety relative to the catalytic residues, whereas longer linkers introduce excessive flexibility, resulting in a broader range of accessible binding orientations. The observed consistency between solvent and no-water conditions suggests that linker length contributes to the overall binding behavior, whereas solvent effects primarily modulate the energetic landscape without altering the general trends observed across the series. It should be noted that the predicted binding energy differences among the conjugates are relatively small and fall within the reported uncertainty of empirical docking scoring functions such as AutoDock Vina v1.2.7. Therefore, the docking scores should be interpreted qualitatively to identify overall trends rather than to establish statistically significant differences between individual linker lengths. Accordingly, the docking results were interpreted together with the geometric analysis of the docked poses, particularly the ester bond–histidine distances, to provide complementary structural insight into the predicted catalytic accessibility.

To complement the initial docking-score comparison, the top-ranked C6 and C12 conformations obtained during the initial screening were examined using UCSF Chimera. These conjugates were selected as representative intermediate- and long-linker systems for subsequent structural and molecular dynamics analyses. The spatial distance between a predefined oxygen atom within the cleavable ester region and the catalytic histidine residue was measured as a comparative descriptor of proximity to the catalytic region.

**Figure 2:**
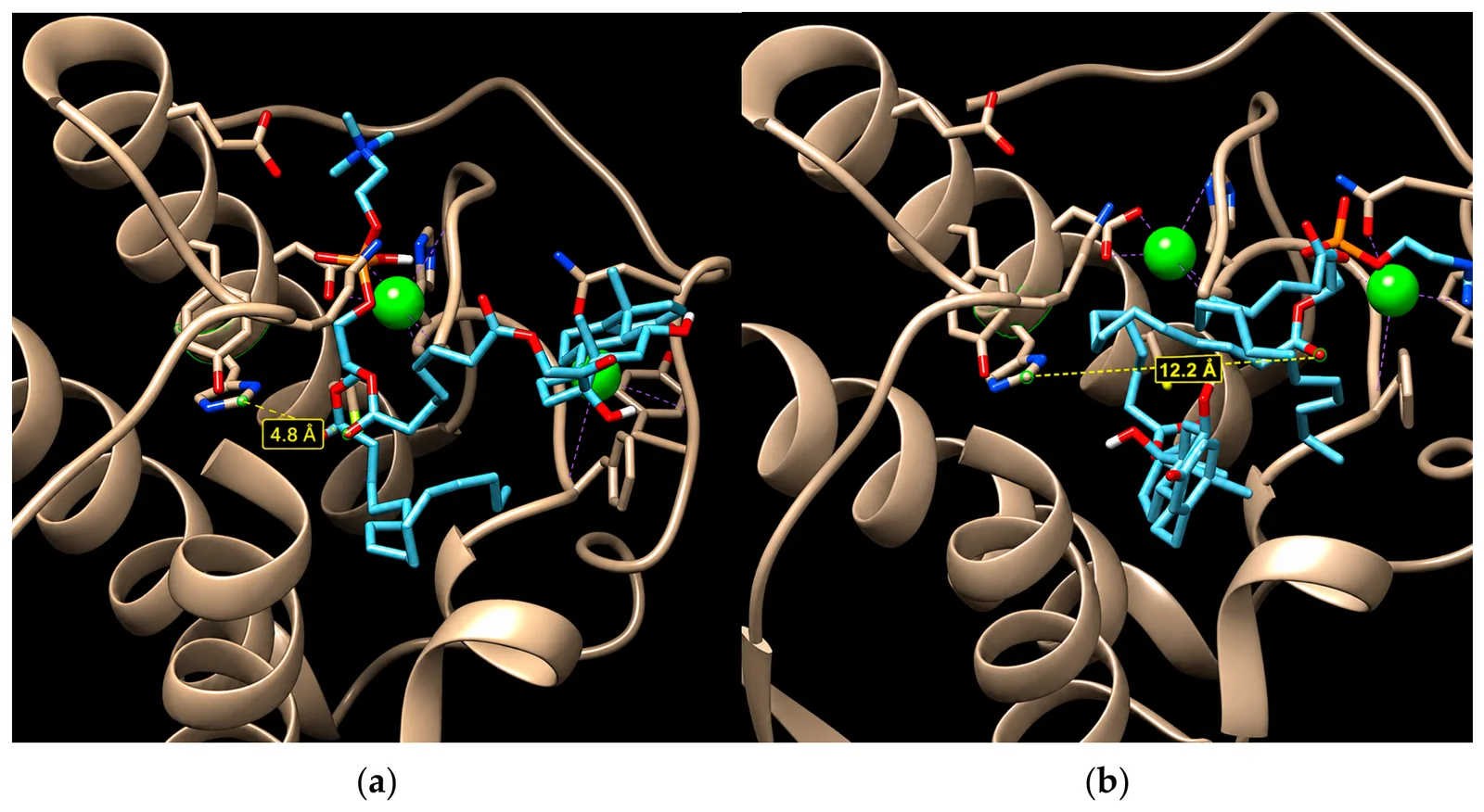
Representative conformations obtained during the initial docking screening of the LPC-C6-PR (**5b**)- and LPC-C12-PR conjugates in the active site of human sPLA_2_ using an exhaustiveness value of 8. (**a**) LPC-C6-PR (**5b**)-conjugate showing an ester-region–histidine distance of 4.8 Å. (**b**) LPC-C12-PR conjugate showing a corresponding distance of 12.2 Å. These distances were used as comparative geometric descriptors of catalytic-site proximity and do not independently establish a catalytically productive geometry. The protein is shown in cartoon representation, the conjugates are displayed as sticks, the catalytic Ca^2+^ ion is shown as a green sphere, and the measured distances are indicated by yellow dashed lines.

In the initial docking poses, the C6-linked conjugate exhibited an ester-region–histidine distance of 4.8 Å, whereas the corresponding distance for the C12-linked conjugate was 12.2 Å (Figure 2). These initial observations suggested that C6 was positioned closer to the catalytic region than C12 and therefore motivated the selection of these two contrasting conjugates for a more extensive conformational search.

The measured distances were interpreted only as descriptors of catalytic-site proximity. Because histidine functions as a general base rather than as a direct nucleophile, a scalar distance alone does not demonstrate a hydrolysis-competent configuration, which additionally depends on angular orientation, calcium coordination, water positioning, and other active-site interactions.

##### Refined Docking Analysis of the C6 and C12 Conjugates

To assess the robustness of the initial docking observations and to improve conformational sampling, the C6- and C12-linked conjugates were subjected to a second, focused docking analysis using an increased exhaustiveness value of 64. The dehydrated 1POE receptor, grid center, grid dimensions, and receptor-preparation protocol were maintained unchanged. Nine poses were generated and retained for each conjugate.

For C6, the predicted binding affinities ranged from −6.9 to −6.3 kcal/mol. The top-ranked pose, Mode 0, displayed a predicted binding affinity of −6.9 kcal/mol and distances of 8.19 Å and 3.15 Å from the predefined ester-region oxygen atom to the histidine Nδ1 atom and the catalytic Ca^2+^ ion, respectively. Mode 1 exhibited somewhat less favorable predicted affinity (−6.5 kcal/mol) but shorter corresponding distances of 7.78 Å and 2.82 Å.

For C12, the predicted binding affinities ranged from −7.0 to −6.7 kcal/mol. Modes 0 and 1 were co-ranked with predicted affinities of −7.0 kcal/mol but displayed markedly different geometries. Mode 0 positioned the predefined ester-region oxygen 13.20 Å from the histidine Nδ1 atom and 7.12 Å from Ca^2+^, whereas Mode 1 positioned the same atom substantially closer to the catalytic region, at distances of 7.84 Å and 2.59 Å, respectively. Additional C12 poses, including Modes 5 and 6, also approached the catalytic region, although they received slightly less favorable docking scores.

The refined analysis therefore revealed catalytically proximal conformations for both linker lengths. Although the initial screening indicated a marked geometric advantage for C6, the more extensive conformational search showed that C12 can also adopt poses positioned near the catalytic histidine and Ca^2+^ ion. Thus, the refined static docking results did not establish a clear geometric advantage for C6 over C12. Instead, they suggested that linker elongation increases the range of accessible conformations and that affinity ranking alone is insufficient for selecting a mechanistically relevant pose.

Distances were measured from the same predefined ester-region oxygen atom to the selected histidine Nδ1 atom and the catalytic Ca^2+^ ion. These distances describe spatial proximity and should not be interpreted independently as evidence of catalytic competence. Complete results for all nine poses of each conjugate are provided in Table 1.

##### Refined Structural and Electrostatic Analysis of the C6 and C12 Conjugates

To complement the refined docking-score and distance analyses, representative structural and electrostatic surface views were generated for the C6 and C12 conjugates. The C6 PyRx Mode 0 pose was selected because it represented the top-ranked C6 conformation, with a predicted binding affinity of −6.9 kcal/mol. In this pose, the predefined ester-region oxygen was positioned 8.19 Å from the selected histidine Nδ1 atom and 3.15 Å from the catalytic Ca^2+^ ion. For C12, PyRx Mode 1 was selected because it was co-ranked with Mode 0 at −7.0 kcal/mol while exhibiting substantially greater proximity to the catalytic region, with corresponding distances of 7.84 Å and 2.59 Å, respectively (Table 2).

The structural representations illustrate that both conjugates can adopt conformations extending toward the catalytic region of human sPLA_2_. The refined C12 pose demonstrates that the longer linker is not intrinsically excluded from the catalytic site, despite the distal orientation observed for C12 during the initial docking screen. Nevertheless, the two conjugates display distinct spatial arrangements of their linker and prednisolone moieties within and around the enzyme-binding cavity.

**Figure 3:**
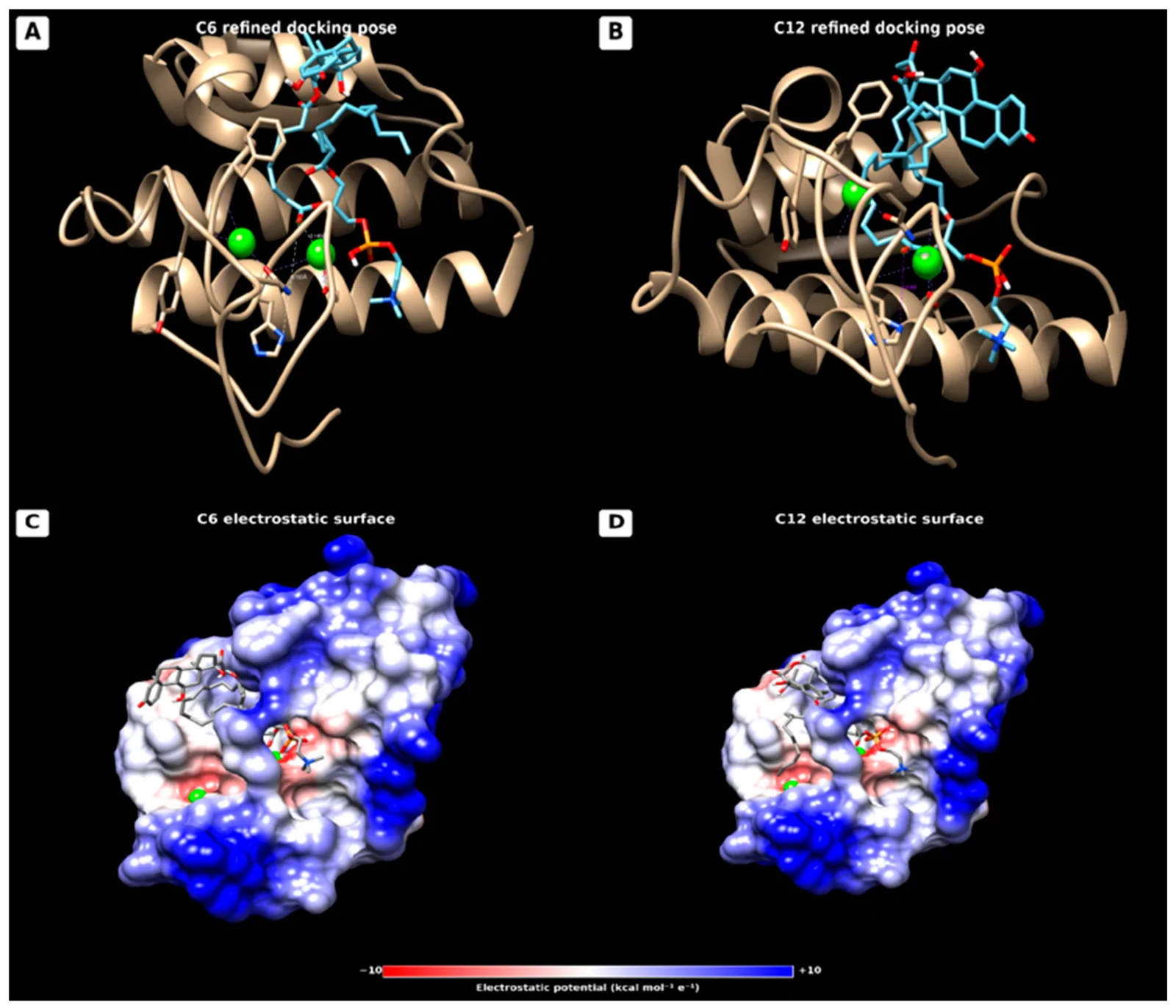
Refined structural and electrostatic surface analysis of the C6- and C12-linked LPC–prednisolone conjugates in complex with human secretory phospholipase A_2_. Docking was performed using AutoDock Vina v1.2.7 with an exhaustiveness value of 64. (**A**) Structural representation of the C6 PyRx Mode 0 pose, with a predicted binding affinity of −6.9 kcal/mol. The predefined ester-region oxygen was positioned 8.19 Å from the selected histidine Nδ1 atom and 3.15 Å from the catalytic Ca^2+^ ion. (**B**) Structural representation of the C12 PyRx Mode 1 pose, with a predicted binding affinity of −7.0 kcal/mol and corresponding distances of 7.84 and 2.59 Å, respectively. (**C**,**D**) Electrostatic surface representations of the C6 and C12 complexes, respectively. The molecular surface was colored according to the calculated Coulombic potential using a scale from −10 kcal/(mol·e), shown in red, through 0, shown in white, to +10 kcal/(mol·e), shown in blue. The conjugates are displayed as sticks, and the catalytic Ca^2+^ ions are shown as green spheres. The measured distances and surface representations are comparative structural descriptors and do not independently demonstrate a hydrolysis-competent configuration. Dashed lines indicate the measured distances from the predefined ester-region oxygen atom to His47 and the catalytic Ca^2+^ ion.

Electrostatic surface representations further illustrated the accommodation of the C6 and C12 conjugates within the heterogeneous electrostatic environment surrounding the catalytic region (Figure 3). Both ligands extended across surface regions containing positive, negative, and near-neutral electrostatic potentials, although their spatial trajectories across the protein surface differed. These views provide a qualitative representation of cavity occupation and ligand accommodation but were not used as quantitative evidence of binding strength or catalytic competence.

Overall, the refined docking and surface analyses demonstrate that both linker lengths can generate catalytic-region-proximal poses. Consequently, the static docking results alone do not establish a clear geometric advantage for C6 over C12. The predicted differences between the two representative conjugates became more apparent in the subsequent molecular dynamics analysis, which evaluates the persistence and stability of their orientations over time. Because molecular dynamics simulations were subsequently performed only for these two representative conjugates, the dynamic behavior of the remaining linker lengths was not evaluated in the present study.

The same predefined ester-region oxygen atom was used for all measurements. The distances represent comparative descriptors of catalytic-site proximity and do not independently establish catalytic competence.

#### 2.2.2. Molecular Dynamics Simulations

**Figure 4:**
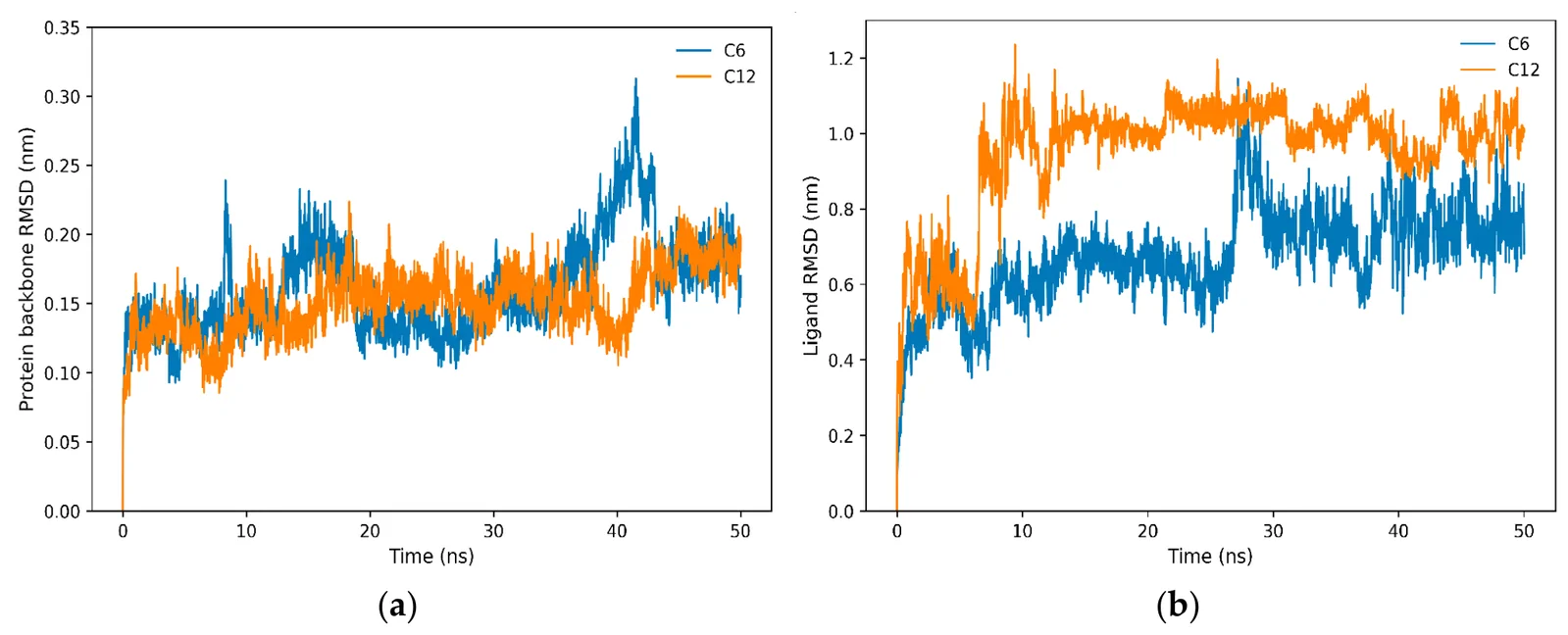
Structural stability of the human sPLA_2_ complexes with the C6 and C12 phospholipid–prednisolone conjugates during 50 ns molecular dynamics simulations. (**a**) Root-mean-square deviation of the protein backbone and (**b**) root-mean-square deviation of the conjugates following alignment of the trajectories to the protein.

To further investigate the stability and dynamic behavior of the phospholipid–prednisolone conjugates in complex with human sPLA_2_, 50 ns molecular dynamics simulations were performed for the C6 and C12 derivatives. The protein backbone RMSD profiles demonstrated that both complexes remained stable throughout the simulation period (Figure 4a). After an initial equilibration phase, the RMSD values fluctuated within a relatively narrow range, indicating that the overall structure of sPLA_2_ was preserved in both systems. Although transient fluctuations were observed, no major conformational changes occurred during the simulations.

The ligand RMSD analysis revealed distinct dynamic behaviors for the two conjugates (Figure 4b). The C12 conjugate exhibited consistently higher RMSD values than the C6 conjugate, suggesting increased mobility within the binding site. In contrast, the C6 conjugate maintained lower RMSD values throughout most of the simulation, indicating comparatively greater conformational stability within the binding site.

**Figure 5:**
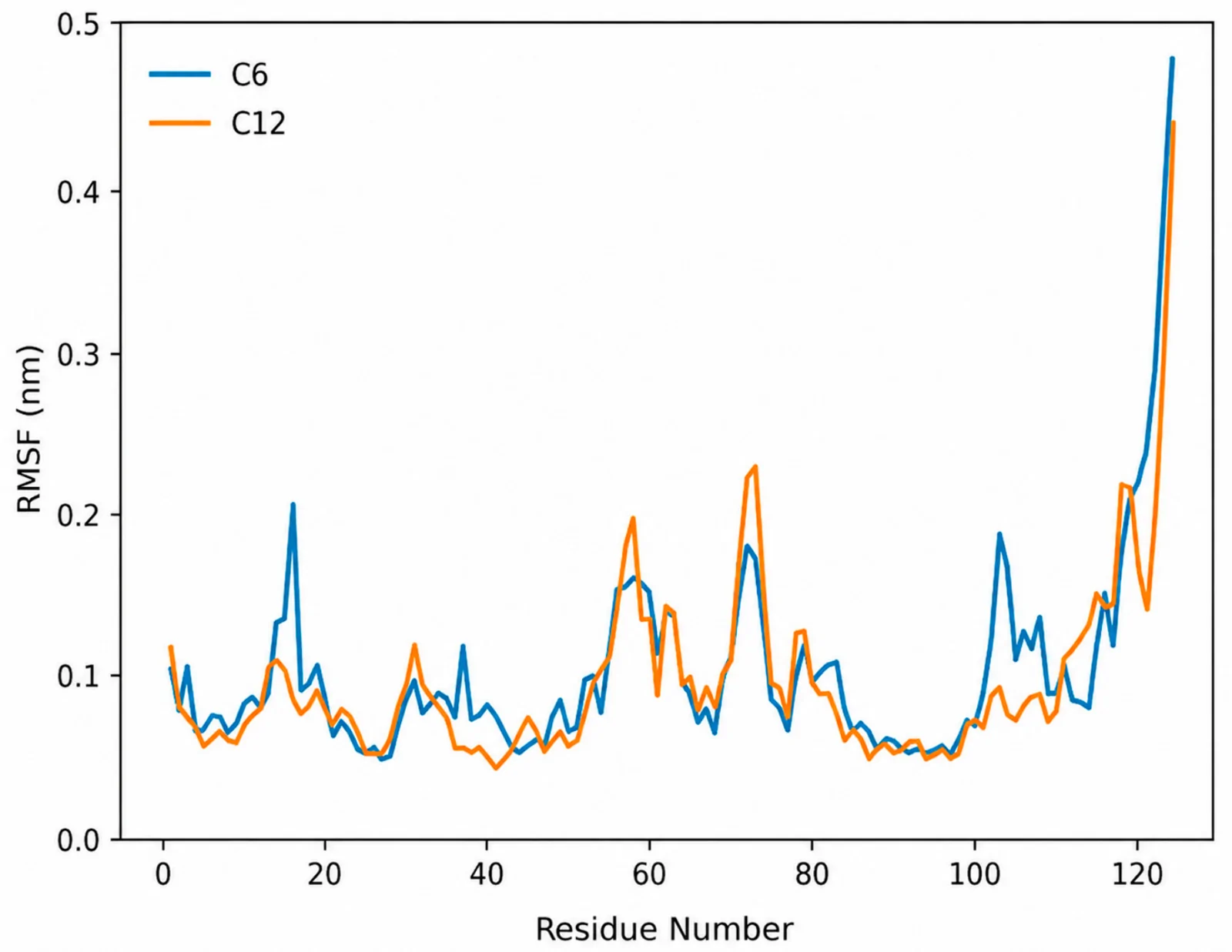
Root-mean-square fluctuation (RMSF) profiles of PLA_2_ residues during 50 ns molecular dynamics simulations of the phospholipid–prednisolone conjugates containing C6 and C12 linkers. The RMSF values were calculated for all protein residues to evaluate the effect of linker length on the flexibility and dynamic behavior of the enzyme–prodrug complexes.

The RMSF profiles of the protein residues were generally similar for the two complexes (Figure 5). Nevertheless, local differences in residue flexibility were observed, suggesting that linker length may influence the dynamic behavior of specific regions of sPLA_2_. These differences may reflect variations in the interactions and spatial organization of the phospholipid–prednisolone conjugates within the enzyme binding site.

**Figure 6:**
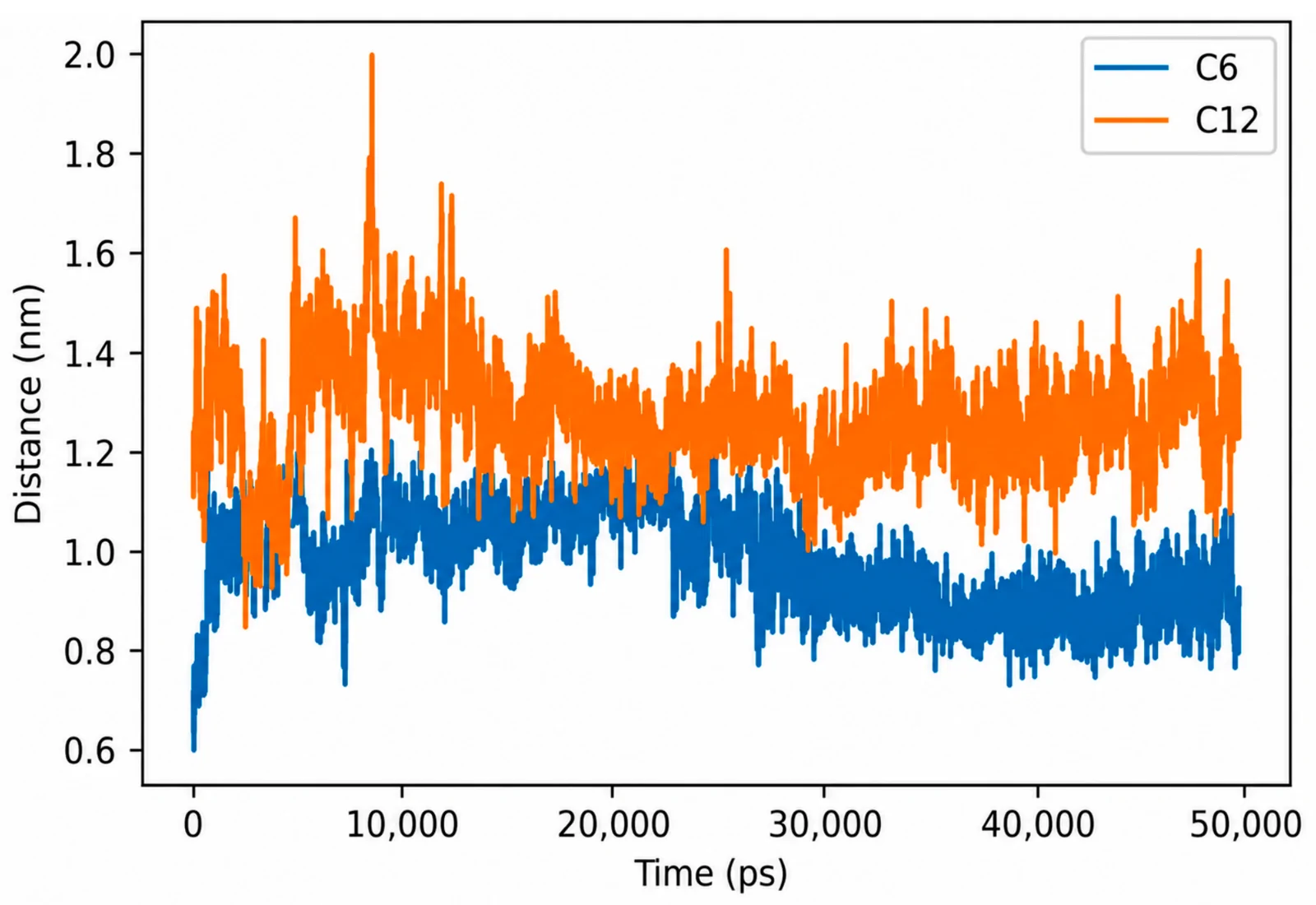
Distance between the predefined ester-region oxygen atom and the catalytic residue His47 during 50 ns molecular dynamics simulations. The measured distances represent comparative geometric descriptors of catalytic-site proximity and do not independently establish catalytic competence.

To further evaluate the relative proximity of the ester region to the catalytic site, the distance between the ester bond of the phospholipid–prednisolone conjugates and the catalytic residue His47 was monitored during the simulations (Figure 6). The C6 conjugate maintained a shorter average distance to His47 compared with the C12 conjugate. The average distances were approximately 0.98 nm and 1.27 nm for C6 and C12, respectively. These findings suggest that, among the two representative conjugates examined by molecular dynamics, the C6 linker maintained a comparatively shorter average distance between the ester region and His47 than the C12 linker. This observation is consistent with more persistence of the ester region in proximity to the catalytic site during the simulations. However, the measured distances represent comparative geometric descriptors and should not be interpreted as direct evidence of a hydrolysis-competent configuration or efficient enzyme-mediated hydrolysis. Because molecular dynamics simulations were performed only for the representative C6 and C12 conjugates, these observations should not be interpreted as describing the dynamic behavior of the complete linker series.

## 3. Discussion

The present study aimed to investigate the effect of linker length on the structural and theoretical activation properties of LPC–prednisolone conjugates designed as phospholipid-based prodrugs for targeted IBD therapy. This approach leverages the high expression of secretory phospholipase A2 (sPLA_2_) in the inflamed intestinal mucosa, which serves as a site-specific trigger for drug release [28]. By combining synthetic chemistry with in silico modeling, we sought to elucidate how variations in spacer length influence binding behavior, conformational stability, and catalytic feasibility within the active site of human sPLA_2_. The study was designed to establish a structurally defined series of novel phospholipid–prednisolone conjugates and to systematically evaluate the influence of linker length on their predicted interaction with human sPLA_2_. Accordingly, synthesis, structural characterization, molecular docking, molecular dynamics simulations, and geometric analysis were integrated to identify linker-dependent trends in catalytic accessibility and predicted activation behavior.

The initial docking screening, performed with an exhaustiveness value of 8, suggested a linker-length-dependent trend in predicted binding behavior. Intermediate linker lengths, particularly C4–C6, exhibited more favorable predicted binding affinities than the longer spacers. Based on the combined energetic and geometric profiles obtained during this initial screening, the C6- and C12-linked conjugates were selected as representative favorable and unfavorable systems, respectively, for more detailed analysis. In the initially selected poses, C6 displayed substantially closer positioning of the cleavable ester region toward the catalytic histidine residue than C12.

However, the subsequent refined docking analysis performed with an increased exhaustiveness value of 64 provided a more nuanced description of the conformational behavior of these two conjugates. The expanded conformational search identified poses positioned near the catalytic histidine residue and the catalytic Ca^2+^ ion for both C6 and C12. In particular, one of the co-top-ranked C12 poses exhibited catalytic-region proximity comparable to that observed for C6. Therefore, the refined static docking analysis did not establish a clear geometric advantage for C6 over C12. Instead, it demonstrated that both linker lengths can adopt conformations approaching the catalytic region, while also revealing that energetically similar docking poses may exhibit substantially different spatial arrangements.

The distinction between the two conjugates became more apparent when the docking results were considered together with the 50 ns molecular dynamics simulations. The protein backbone remained stable in both complexes, whereas the C12 conjugate exhibited higher ligand RMSD values than C6, indicating greater mobility within the binding site. In contrast, C6 maintained a more stable orientation relative to the enzyme. Thus, although both conjugates were capable of adopting catalytic-region-proximal poses during docking, their ability to retain such positioning under dynamic conditions appeared to differ. It should be noted, however, that these conclusions are based only on the C6 and C12 conjugates, which were selected as representative linker lengths for molecular dynamics analysis. Therefore, the dynamic behavior of the remaining conjugates cannot be inferred from the present results.

From a mechanistic perspective, these findings may be explained by the interplay between conformational flexibility, structural preorganization, and the catalytic requirements of sPLA_2_. Productive positioning of the sn-2 ester bond within the catalytic region of the enzyme is considered an important prerequisite for efficient hydrolysis, where substrate organization is influenced by active-site residues, the catalytic Ca^2+^ ion, water-mediated interactions, and the angular orientation of the reacting groups [24]. Within this catalytic mechanism, the catalytic histidine functions as a general base rather than as a direct nucleophile. Consequently, the measured ester-region–histidine and ester-region–Ca^2+^ distances should be interpreted as comparative descriptors of catalytic-site proximity rather than as direct evidence of a hydrolysis-competent configuration.

The refined docking results demonstrate that both C6 and C12 can adopt conformations approaching the catalytic region. However, the occurrence of an individual proximal docking pose does not establish that this orientation will remain stable under dynamic conditions. Intermediate linker lengths may provide an advantageous balance between rigidity and flexibility, allowing accommodation of the prednisolone moiety while limiting excessive conformational freedom [29]. In contrast, elongation of the linker introduces additional rotational degrees of freedom and broadens the conformational ensemble accessible to the conjugate. This increased flexibility does not necessarily prevent access to the catalytic region, as demonstrated by the proximal C12 poses obtained during refined docking, but it may reduce the persistence of a potentially favorable orientation.

The electrostatic surface representations provide additional qualitative insight into the accommodation of the conjugates within the sPLA_2_ binding region. Both C6 and C12 extended across a heterogeneous surface containing positively charged, negatively charged, and near-neutral regions, while adopting distinct spatial arrangements of their linker and prednisolone moieties. These observations support the conclusion that both linker lengths can be accommodated within or adjacent to the catalytic-region cavity. However, the electrostatic surface maps should not be interpreted as quantitative measures of electrostatic complementarity or binding strength, because no energetic decomposition or electrostatic interaction-energy calculation was performed. Their primary purpose was to visualize cavity occupation and the relative spatial organization of the docked conjugates.

Redocking of the co-crystallized GEL ligand reproduced the experimentally observed binding geometry. The top-ranked pose displayed a symmetry-corrected heavy-atom RMSD of 1.89 Å relative to the crystallographic conformation, based on 31 heavy atoms. This value is below the commonly applied 2.0 Å threshold and supports the ability of the docking procedure to recover a crystallographically consistent binding pose.

Consistent with this interpretation, RMSF analysis revealed generally similar fluctuation profiles for the C6 and C12 complexes, although local differences in residue flexibility were observed, suggesting that linker length affects the dynamic behavior and spatial organization of specific regions within the enzyme. Moreover, trajectory-based distance analysis demonstrated that C6 maintained a shorter average distance between the ester region and the catalytic residue His47 than C12, with average values of approximately 0.98 and 1.27 nm, respectively. Taken together, these findings suggest that, among the two linker lengths examined by molecular dynamics, C6 appeared to maintain greater persistence and conformational stability in its catalytic-region positioning than C12, rather than providing exclusive access to the active site. However, because molecular dynamics simulations were performed only for these two representative conjugates, these observations should not be interpreted as establishing an optimal linker length for the entire series. Nevertheless, the trajectory-based distances remain geometric descriptors and do not independently establish catalytic competence [24,30].

These observations are consistent with previous studies on phospholipid-based prodrugs, which suggested that linker optimization helps balance steric accessibility and conformational freedom within the PLA_2_ active site. Computational and experimental investigations have shown that linker elongation initially may improve the accessibility of bulky drug moieties by distancing them from the phospholipid backbone; however, beyond a certain linker length, additional methylene units may primarily increase molecular flexibility without necessarily providing additional catalytic advantage [31,32].

Specifically, studies on diclofenac and indomethacin conjugates suggested that relatively short linkers (4–6 methylene units) are associated with more favorable catalytic alignment [33,34], whereas larger and more structurally complex drugs, such as cyclosporine, may benefit from longer spacers to achieve favorable positioning within the enzyme active site [35]. In contrast to the phospholipid–cyclosporine system, in which longer linkers improved enzymatic activation, the present results suggest that the smaller and less sterically demanding prednisolone moiety may benefit from intermediate linker lengths. Collectively, these findings support the concept that successful PLA_2_-responsive prodrug design requires careful optimization of linker architecture, balancing sufficient flexibility for enzyme accommodation with adequate structural preorganization to maintain favorable positioning under dynamic conditions.

Importantly, in these previous approaches, the synthetic strategy typically involved initial coupling of the drug to the linker, followed by attachment to the phospholipid backbone. In contrast, the present study employs a reversed synthetic strategy in which the phospholipid–linker intermediate is generated prior to drug conjugation. This modular approach separates scaffold construction from payload incorporation, facilitating the future incorporation of structurally diverse therapeutic agents while preserving the same phospholipid-based delivery platform.

Beyond the synthetic innovation, the present work extends our phospholipid–linker platform to prednisolone, a corticosteroid that differs substantially from previously investigated drugs in terms of molecular structure, physicochemical properties, and therapeutic application. Whereas our earlier studies focused on NSAIDs and cyclosporine, the current study addresses corticosteroid delivery for inflammatory bowel disease, thereby broadening the applicability of the platform and providing new insights into linker optimization for structurally distinct therapeutic agents. Accordingly, the present study should be viewed as an extension and validation of the phospholipid–linker platform for a new therapeutic class rather than as a direct repetition of our previous work.

Such modularity may help systematic structure–activity investigations across different drug classes. It can also enable more efficient optimization of linker composition, molecular orientation, and activation profiles. In addition, maintaining a common phospholipid scaffold may improve comparability between conjugates and support broader platform development for enzyme-responsive lipid-based prodrugs.

From a broader perspective, this study highlights the importance of rational linker design in the development of enzyme-responsive prodrugs. Results emphasize that linker length does not merely control the distance between the drug and the phospholipid carrier, but also influences the range of accessible conformations, ligand mobility, and the persistence of favorable positioning within the catalytic region. These structural insights may contribute to the future design and optimization of phospholipid-based delivery systems targeting inflammatory conditions such as IBD.

Despite these promising findings, several limitations should be considered. First, molecular dynamics simulations were performed only for the C6 and C12 conjugates, selected as representative linker lengths based on the initial docking analysis. Consequently, the dynamic behavior of the remaining linker lengths was not evaluated and cannot be generalized from the present results. Future studies, including molecular dynamics simulations across the entire linker series, will provide a more comprehensive understanding of linker-length-dependent behavior. In addition, molecular docking and geometric analysis provide predictive rather than quantitative evidence of enzyme–ligand interactions. The relatively small differences in AutoDock Vina scores fall within the reported uncertainty of empirical docking scoring functions. Therefore, the docking scores should be interpreted qualitatively and together with structural and dynamic analyses rather than as definitive measures of linker preference. Moreover, the refined docking results demonstrated that poses with similar predicted affinities may differ substantially in their proximity to the catalytic region, emphasizing that affinity ranking alone is insufficient for identifying a mechanistically relevant conformation.

Furthermore, the electrostatic surface representations were used for qualitative visualization only and do not provide a quantitative estimate of electrostatic binding contributions.

In addition, the identification of catalytic-region-proximal poses does not demonstrate that the conjugates adopt a complete hydrolysis-competent geometry. The present docking analysis did not quantitatively evaluate reaction angles, proton-transfer geometry, the orientation of the catalytic water molecule, or the complete network of calcium-mediated interactions. The measured distances to the catalytic histidine and Ca^2+^ ion should therefore be regarded as comparative spatial descriptors. Although molecular dynamics simulations provide additional information regarding conformational stability and persistence, the 50 ns trajectories do not directly simulate the chemical hydrolysis reaction. Furthermore, the present MD analysis was designed as an exploratory structural investigation, focusing on global stability, residue flexibility, ligand mobility, and catalytic-site proximity rather than exhaustive conformational sampling. Consequently, the 50 ns simulations should not be considered fully converged, and longer simulation times and/or multiple independent simulation replicas would be required to more comprehensively characterize the conformational landscape and capture rare but potentially catalytically relevant conformations. More detailed mechanistic analyses, including catalytic-angle evaluation, catalytic water-network analysis, conformational-state population analysis, and free-energy calculations, were beyond the scope of the present study. Accordingly, the molecular dynamics results should be interpreted as hypothesis-generating structural evidence rather than as definitive mechanistic proof of PLA_2_-catalyzed hydrolysis.

Other factors, including membrane interactions, enzyme dynamics, ligand protonation states, and local microenvironmental conditions, may also substantially influence prodrug recognition and activation in biological systems [32]. Experimental PLA_2_-mediated cleavage assays will therefore be required to determine whether the predicted differences in conformational stability and catalytic-region positioning translate into measurable differences in enzymatic activation.

Future studies should therefore focus on experimental validation of the predicted trends, including in vitro enzymatic activation assays and cell-based models of inflammation. Further investigation into membrane interactions and prodrug stability in biological media would also provide valuable insights into the translational potential of this platform. Moreover, expanding this modular synthetic approach to additional therapeutic agents may help establish general design principles for phospholipid-based prodrugs.

## 4. Materials and Methods

### 4.1. Chemistry

#### General

All reagents were obtained from commercial suppliers and used without further purification. Reaction progress was monitored by thin-layer chromatography (TLC), ^1^H-NMR and LC-MS. Dichloromethane was dried using a Bio-LAB Ltd. solvent purification system (Bio-Lab Ltd., Jerusalem, Israel). Analytical thin-layer chromatography (TLC) was performed using 250 μm Silica Gel 60 F_254_ pre-coated plates. Polymeric reversed-phase Strata^TM^ -X cartridges (33 µm; Phenomenex, Torrance, CA, USA) were used for purification. Proton nuclear magnetic resonance (^1^H NMR) spectra were recorded using a Bruker AVANCE III 500 MHz spectrometer (11.7 T; Bruker BioSpin GmbH, Ettlingen, Germany) at Ben-Gurion University. Chemical shifts (δ) are reported in parts per million (ppm) downfield relative to tetramethylsilane (TMS, 0.0 ppm), CDCl_3_ (7.27 ppm), CD_3_OD (3.31 ppm), (CD_3_)_2_SO (2.50 ppm). Coupling constants (J) are reported in Hz. Multiplicities are reported using the following abbreviations: s, singlet; d, doublet; t, triplet; q, quartet; p, pentet; m, multiplet; b, broad.

Carbon-13 nuclear magnetic resonance (^13^C NMR) spectra were recorded using a Bruker AVANCE III 500 MHz spectrometer (11.7T) and a Bruker Avance Neo 400 MHz (Bruker BioSpin GmbH, Ettlingen, Germany) at Ben-Gurion University. Chemical shifts are reported in ppm relative to the carbon resonance of CDCl_3_ (77.16 ppm), CD_3_OD (49.0 ppm), (CD_3_)_2_SO (39.52 ppm). Liquid chromatography-mass spectra (LC-MS) were obtained using an Orbitrap Exploris 240 mass spectrometer (Thermo Fisher Scientific, Bremen, Germany) equipped with an electrospray ionization source at Ben-Gurion University and are reported as *m*/*z* (relative abundance). Data acquisition was performed in full-scan positive and negative ion modes at a resolution of 24,000 full width at half-maximum (FWHM). Data acquisition and analysis were performed using Xcalibur software, v4.5 (Thermo Scientific, Waltham, MA, USA).

General procedure for prednisolone coupling (B): N,N′-dicyclohexylcarbodiimide (DCC, ~0.45 mmol, 1.5 equiv), DMAP (~0.6 mmol, 2 equiv) and prednisolone 4 (~0.3 mmol, 1.0 equiv) were added to a solution of compound 3a–d (~0.3 mmol, 1 equiv) in distilled dichloromethane (30 mL) at room temperature. After stirring at RT for 48 h, the reaction mixture was evaporated, and the residue was dissolved in MeOH. The products were purified by HPLC using a gradient of IPA:MeOH/water, affording the desired products.

*LPC-C4-PR (LPC-4-Butyric acid-prednisolone-conjugate)* **(5a):** The title compound was prepared via procedure (B) with compound 3a (200 mg, 0.32 mmol), DCC (99 mg, 0.48 mmol), DMAP (78 mg, 0.64 mmol) and prednisolone (115 mg, 0.32 mmol) in dichloromethane (30 mL) at RT for 48 h. Purification by HPLC using IPA:MeOH/water afforded the desired product 5a as a white solid (164 mg, 24.5%).; ^1^H NMR (500 MHz, CDCl_3_) δ 6.29 (d, *J* = 1.40 Hz, 1H), 6.03 (s, 1H), 5.00 (s, 1H), 4.96 (s, 1H), 4.92 (s, 1H), 4.89 (s, 1H), 4.49 (d, *J* = 2.66 Hz, 1H), 3.43–3.35 (m, 4H), 2.78–2.69 (m, 1H), 2.58 (s, 1H), 2.49 (d, *J* = 4.38 Hz, 1H), 2.35 (dt, *J* = 4.37,2.82 Hz, 1H), 1.94(d, *J* = 12.51,3.07 Hz, 10H),1.86–1.78 (m, 1H), 1.74 (dt, *J* = 13.22, 3.93 Hz, 14H), 1.62 (d, *J* = 13.05 Hz, 5H), 1.46 (s, 7H), 1.37–1.30 (m, 10H), 1.24–1.15 (m, 16H), 1.11–1.05 (m, 2H),0.97 (s, 4H). ^13^C NMR (500 MHz, CDCl_3_) δ205.88, 157.74, 157.14, 128.89, 123.48, 90.72, 71.1, 68.96, 56.33, 52.39, 50.20, 48.87, 40.47, 35.66, 35.03, 34.93, 34.80, 34.37, 32.96, 32.27, 30.71, 26.60, 25.93, 25.75, 25.12, 24.85, 22.08, 17.94, 0.99. HRMS (ESI) calcd for C_51_H_84_NO_14_P [M+H]+965.56, found 966.56.

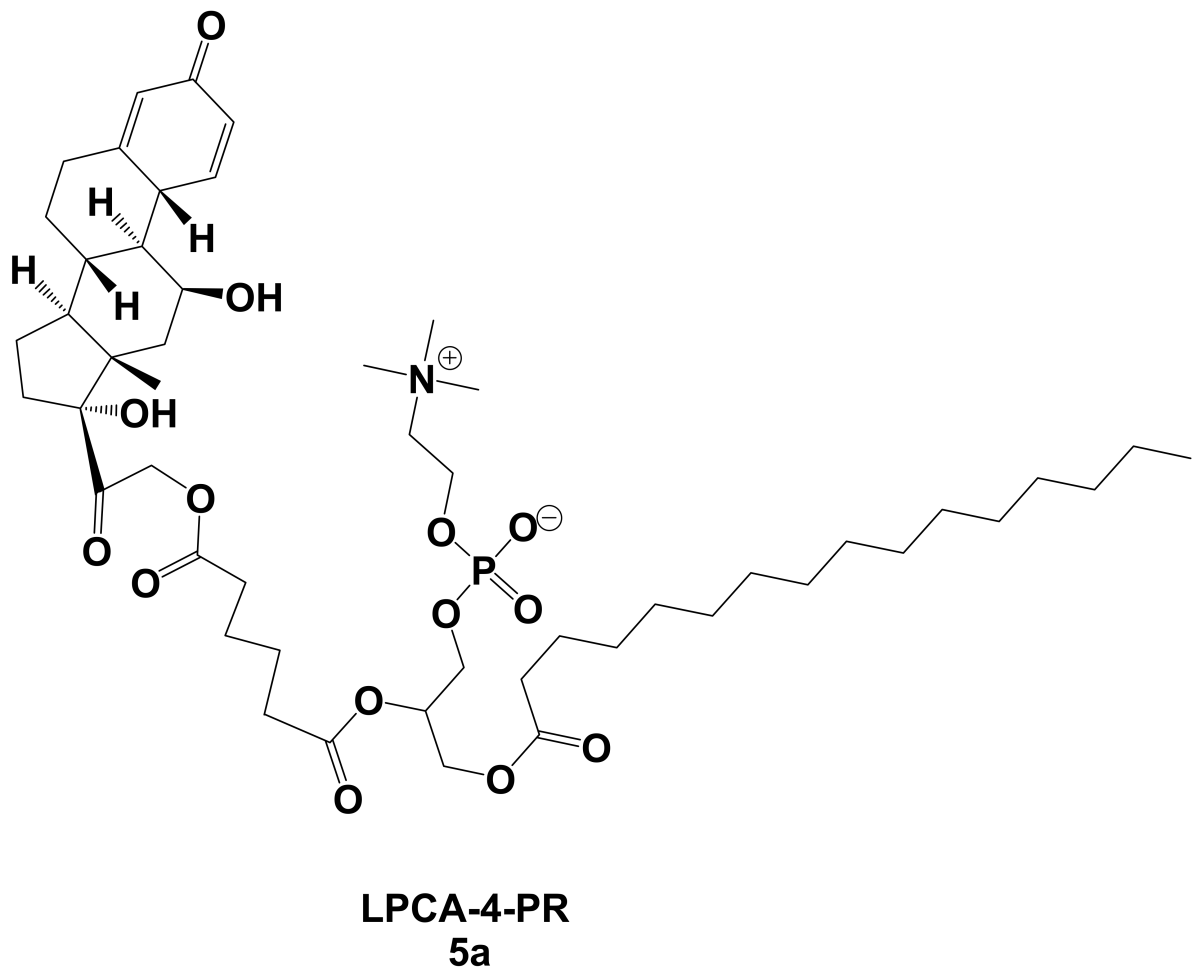

*LPC-C6-PR (LPC-6-hexanoic acid-prednisolone-conjugate)* **(5b):** The title compound was prepared via procedure (B) with compound 3b (200 mg, 0.31 mmol), DCC (95 mg, 0.46 mmol), DMAP (75 mg, 0.62 mmol) and prednisolone (112 mg, 0.31 mmol) in dichloromethane (30 mL) at RT for 48 h. Purification by HPLC using IPA: MeOH/water afforded the desired product 5b as a white solid (105.6 mg, 14.32%).; ^1^H NMR (500 MHz, CDCl_3_) δ 6.30 (m, 1H), 6.05 (q, 1H), 5.13–4.86 (m, 1H), 4.81–4.73 (m, 1H), 4.68–4.61 (m, 1H), 4.52–4.48 (m, 1H), 4.35–4.26 (m, 1H), 3.68 (s, 1H), 3.37 (m, 1H), 3.26 (s, 1H), 2.83–2.65 (m, 1H),2.36 (m, 35H), 1.99–1.90 (m, 5H),1.83–1.71 (m, 6H), 1.66–1.58 (m, 3H), 1.54–1.30 (m, 13H), 1.28–1.05 (m, 13H), 0.98 (s, 2H). ^13^C NMR (400 MHz, CDCl_3_) δ 212.00, 157.96, 156.53, 156.2, 127.89, 127.76, 122.45, 122.37, 70.21, 70.15, 67.79, 67.42, 55.40, 55.27, 51.37, 51.16, 50.06, 39.77, 34.60, 34.23, 34.04, 33.97, 33.67, 33.29, 31.98, 31.27, 29.71,28.33, 25.33, 24.69, 24.48, 23.97, 23.84, 21.05, 17.51, 16.96, -0.02. HRMS (ESI) calcd for C_53_H_88_NO_14_P [M+H]+993.59, found 994.59.

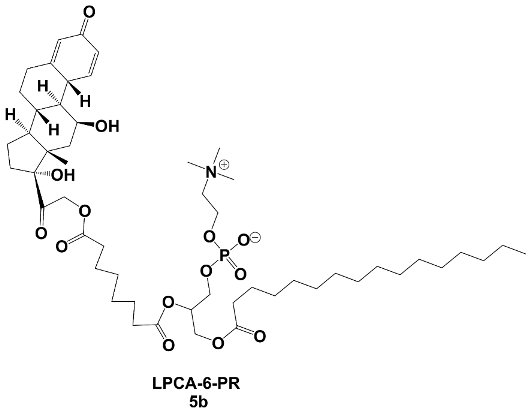

*LPC-C8-PR (LPC-8-oxooctanoic acid-prednisolone-conjugate)* **(5c):** The title compound was prepared via procedure (B) with compound 3c (200 mg, 0.294 mmol), DCC (91 mg, 0.441 mmol), DMAP (72 mg, 0.589 mmol) and prednisolone (106 mg, 0.294 mmol) in dichloromethane (30 mL) at RT for 48 h. Purification by HPLC using IPA:MeOH/water afforded the desired product 5c as a white solid (142.5 mg, 25.22%).; ^1^H NMR (500 MHz, CDCl_3_) δ 6.34 (dt, *J* = 9.90 Hz, 1H), 6.09 (s, 1H), 4.93 (m, 2H), 4.49 (q, *J* = 3.50 Hz, 1H), 2.44 (s, 30H), 2.21–2.00 (m, 5H),1.97–1.87 (m, 2H), 1.86–1.54 (m, 12H),1.53–1.05 (m, 30H), 0.99 (s, 5H), 0.08 (s, 1H), 0.01 (d, *J* = 1.12 Hz, 2H). ^13^C NMR (500 MHz, CDCl3) δ 205.78, 187.84, 174.87, 171.58, 157.68, 128.72, 123.32, 106.77, 90.73, 71.14, 68.83, 56.35, 52.34, 51.14, 48.79, 45.19, 40.49, 35.57, 35.04, 34.97, 34.92, 34.85, 34.23, 33.01, 32.25, 30.72, 29.69, 29.58, 26.30, 25.69, 24.84, 22.03, 17.95. HRMS (ESI) calcd for C_55_H_92_NO_14_P [M+H]+1021.63, found 1022.63.

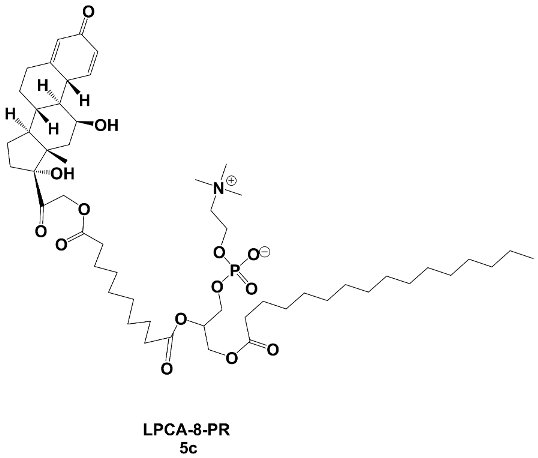

*LPC-C10-PR (LPC-10-oxodecanoic acid-prednisolone-conjugate)* **(5d):** The title compound was prepared via procedure (B) with compound 3d (200 mg, 0.28 mmol), DCC (87 mg, 0.424 mmol), DMAP (70 mg, 0.57 mmol) and prednisolone (101 mg, 0.28 mmol) in dichloromethane (30 mL) at RT for 48 h. Purification by HPLC using IPA:MeOH/water afforded the desired product 5d as a white solid (126.84 mg, 19.74%).; ^1^H NMR (500 MHz, CDCl_3_) δ 6.29 (m, 1H), 6.05 (d, *J* = 1.57 Hz, 1H), 5.42–5.29 (m, 1H), 5.02–4.82 (m, 3H), 4.48 (d, *J* = 2.94 Hz, 1H), 3.67 (s, 1H), 3.45–3.11 (m, 21H), 2.76 (q, *J* = 3.03 Hz, 1H), 2.46–2.41 (m, 2H), 2.38–2.28 (m, 2H), 2.18–2.04 (m, 3H), 1.97–1.91 (m, 4H), 1.81 (t, *J* = 3.96 Hz, 1H), 1.78–1.71 (m, 5H), 1.70–1.59 (m, 7H), 1.52–1.44 (m, 6H), 1.39–1.06 (m, 32H), 0.97 (s, 4H). ^13^C NMR (500 MHz, CDCl_3_) δ205.79, 187.77, 174.92, 171.45, 171.41, 162.35, 158.92, 157.57, 128.88, 128.57, 123.34, 90.79, 90.68, 71.14, 68.88, 56.34, 52.49, 52.48, 52.35, 51.04, 48.78, 45.16, 40.57, 40.50, 35.66, 35.60, 35.22, 35.11, 35.04, 34.87, 34.44, 34.30, 33.00, 32.25, 30.71, 30.58, 30.34, 30.26, 30.18, 30.11, 29.94, 29.86, 29.81, 29.74, 26.33, 25.92, 25.84, 25.71, 24.84, 22.04, 17.95, 0.99. HRMS (ESI) calcd for C_57_H_96_NO_14_P [M+H]+1049.66, found 1050.66.

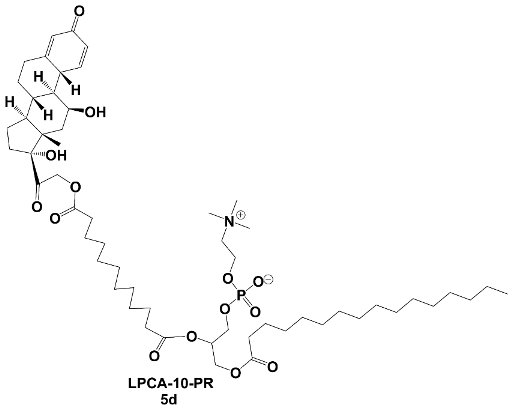


### 4.2. Molecular Modeling

#### 4.2.1. Molecular Docking Simulations

Molecular docking studies were performed to evaluate the predicted binding affinities of phospholipid–prednisolone (PL-prednisolone) conjugates with varying linker lengths toward human secretory phospholipase A_2_ (sPLA_2_). The crystal structure of human secretory PLA_2_ (PDB ID: 1POE) was used as the receptor for docking simulations. Protein preparation was performed using UCSF Chimera by removing non-essential heteroatoms while retaining catalytically relevant calcium ions were retained [36]. Two receptor conditions were evaluated: (i) dehydrated active-site conditions, in which water molecules were removed, and (ii) hydrated conditions, in which structurally relevant water molecules were retained. Polar hydrogens and Gasteiger charges were added, and the prepared protein structures were converted to PDBQT format using PyRx implementing the AutoDock Vina framework [36,37]. The active-site calcium ions were retained throughout the docking simulations because of their catalytic relevance.

A series of six PL-prednisolone conjugates differing in linker length (2, 4, 6, 8, 10, and 12 methylene units) was constructed and geometrically optimized prior to docking analysis. The optimized ligands were converted to PDBQT format using Open Babel implemented within the PyRx platform.

Docking was conducted in two sequential stages. In the initial screening stage, the complete series of six conjugates was docked using AutoDock Vina v1.2.7 [37] with an exhaustiveness value of 8. The docking search space was centered on the catalytic region of human sPLA_2_ and encompassed the catalytic residues and the calcium-binding region. The grid center was set at x = 23.637, y = 7.304, and z = 67.484, with dimensions of 26 × 26 × 26 Å. These dimensions provided coverage of the active-site pocket and sufficient space to accommodate the conjugates irrespective of linker length. Multiple poses were generated and ranked according to their predicted binding affinity. The top-ranked pose of each conjugate was used for the initial comparison of linker-dependent binding behavior.

Based on the combined predicted binding affinity and catalytic-site proximity observed during the initial screening, the C6- and C12-linked conjugates were selected as representative compounds displaying the most favorable and least favorable initial docking profiles, respectively. These two conjugates were subsequently subjected to a focused docking analysis using an increased exhaustiveness value of 64. The refined docking was performed against the dehydrated 1POE receptor while maintaining the same grid center, grid dimensions, and receptor-preparation conditions used in the initial screening. Nine docking poses were generated and retained for each conjugate.

Post-docking structural analyses were performed using UCSF Chimera. For the refined C6 and C12 docking results, all generated poses were evaluated according to both their predicted binding affinity and their spatial proximity to the catalytic region. A predefined oxygen atom within the cleavable ester region was used consistently across all poses. The distances between this atom and the catalytic calcium ion, as well as between this atom and the Nδ1 atom of the catalytic histidine residue, were measured using the built-in structural analysis tools in UCSF Chimera.

The measured distances were used as comparative descriptors of ligand positioning relative to the catalytic region. They were not considered, independently, to demonstrate a hydrolysis-competent configuration, because productive catalysis additionally depends on ligand orientation, water-mediated interactions, and the angular arrangement of the reacting groups. Predicted binding-affinity values obtained from AutoDock Vina were reported in kcal/mol and were interpreted together with the structural-distance analysis.

Electrostatic surface representations were generated for the selected refined docking poses of the C6- and C12-linked conjugates using the Coulombic Surface Coloring tool implemented in UCSF Chimera. The C6 PyRx Mode 0 and C12 PyRx Mode 1 poses obtained using an exhaustiveness value of 64 were visualized on the molecular surface of the dehydrated 1POE receptor. The electrostatic potential was mapped onto the MSMS molecular surface using a three-color scale ranging from −10 kcal/(mol·e) in red, through 0 kcal/(mol·e) in white, to +10 kcal/(mol·e) in blue. A distance-dependent dielectric model was applied with a dielectric constant of 4.0 and a surface-distance parameter of 1.4 Å. Histidine protonation states were estimated according to the hydrogen-bonding environment. Identical surface-calculation parameters, color scales, and receptor coordinates were used for both complexes to permit direct visual comparison.

The docking protocol was validated by redocking the co-crystallized ligand GEL into the binding site of the 1POE structure after removal of the crystallographic ligand. The receptor coordinates were kept fixed, and docking was performed using AutoDock Vina with an exhaustiveness value of 64. The heavy-atom RMSD between the crystallographic and top-ranked redocked poses was calculated without structural fitting, using chemically equivalent atom mapping to account for molecular symmetry.

#### 4.2.2. Molecular Dynamics Simulations

To further investigate the stability and dynamic behavior of two representative the phospholipid–prednisolone conjugates in complex with human secretory phospholipase A_2_ (sPLA_2_), molecular dynamics (MD) simulations were performed using GROMACS 2025.4 [38]. The initial structures of the protein–ligand complexes were obtained from molecular docking.

The simulations were carried out using the AMBER99SB-ILDN force field [39] and the TIP3P water model. Each complex was solvated in a periodic water box and neutralized by the addition of chloride ions. Energy minimization was performed prior to equilibration.

Following minimization, the systems were equilibrated under constant-volume (NVT) and constant-pressure (NPT) conditions for 100 ps each at 300 K and 1 bar. Temperature coupling was achieved using the velocity-rescaling thermostat, whereas pressure coupling was performed using the Parrinello–Rahman barostat.

Production MD simulations were performed for 50 ns using a 2 fs integration time step. Long-range electrostatic interactions were treated using the particle mesh Ewald (PME) method, while van der Waals interactions were calculated using a cutoff distance of 1.0 nm. All bonds involving hydrogen atoms were constrained.

Trajectory analyses were performed using the GROMACS analysis tools. Protein backbone root-mean-square deviation (RMSD), ligand RMSD, and root-mean-square fluctuation (RMSF) analyses were carried out to evaluate the stability and flexibility of the complexes. In addition, the distance between the ester bond of the phospholipid–prednisolone conjugates and the catalytic residue His47 was monitored throughout the simulations to assess the accessibility of the catalytic site.

## 5. Conclusions

In this study, a series of phospholipid–linker–prednisolone conjugates with varying spacer lengths were successfully synthesized using a reversed synthetic strategy and evaluated using molecular docking, structural analysis, and molecular dynamics simulations. Initial docking screening identified the C6- and C12-linked conjugates as representative intermediate- and long-linker systems selected for subsequent detailed computational analysis. However, refined docking with increased conformational sampling demonstrated that both conjugates could adopt poses positioned near the catalytic histidine residue and the catalytic Ca^2+^ ion. The refined static docking analysis therefore did not establish exclusive or clearly superior catalytic-site access for C6.

Molecular dynamics simulations nevertheless revealed differences in the persistence and stability of the predicted binding orientations. C6 exhibited lower ligand mobility and maintained a shorter average distance between the ester region and the catalytic histidine residue than C12. Taken together, these computational findings suggest that, among the two linker lengths examined in detail using molecular dynamics, C6 exhibited greater conformational stability and more persistent positioning relative to the catalytic region than C12. However, these observations should not be interpreted as demonstrating exclusive access to the active site. These results provide structural insights and generate hypotheses that may guide the future optimization of phospholipid-based corticosteroid prodrugs for targeted IBD therapy. However, because molecular dynamics simulations were performed only for the representative C6 and C12 conjugates, the present results should not be interpreted as establishing the optimal linker length for the entire series. Furthermore, the molecular dynamics analysis should be regarded as an exploratory structural investigation rather than a definitive mechanistic characterization of PLA_2_-mediated catalysis. Experimental PLA_2_-mediated cleavage studies, together with more comprehensive computational analyses, will be required to validate the predicted activation behavior and further investigate linker-dependent dynamics.

## Figures and Tables

**Table 1:** Summary of representative poses obtained from refined C6 and C12 docking using an exhaustiveness value of 64.

Conjugate	Mode	Affinity (kcal/mol)	Ester O–His Nδ1 (Å)	Ester O–Ca^2+^ (Å)	Interpretation
C6	0	−6.9	8.193	3.146	Top-ranked C6 pose
C6	1	−6.5	7.782	2.815	Most proximal C6 pose
C12	0	−7.0	13.195	7.119	Co-top-ranked, distal pose
C12	1	−7.0	7.839	2.593	Co-top-ranked, proximal pose
C12	5	−6.8	7.734	2.646	Shortest His distance
C12	6	−6.7	7.802	2.926	Additional proximal pose

**Table 2:** Representative refined docking poses selected for structural and electrostatic analysis.

Conjugate	PyRx Mode	Affinity (kcal/mol)	O–His Nδ1 (Å)	O–Ca^2+^ (Å)	Selection Rationale
C6	0	−6.9	8.193	3.146	Top-ranked C6 pose
C12	1	−7.0	7.839	2.593	Co-top-ranked and catalytically proximal C12 pose

## Data Availability

The original contributions presented in this study are included in the article/Supplementary Materials. Further inquiries can be directed to the corresponding author.

## Supplementary Materials

The following supporting information can be downloaded at https://www.mdpi.com/article/10.3390/molecules31173057/s1. The Supplementary Materials contain NMR spectra and embedded chemical structures.

## Abbreviations

The following abbreviations are used in this manuscript:

IBD	Inflammatory bowel disease
PLA_2_	phospholipase A_2_
LPC	lysophosphatidylcholine
DMAP	4-dimethylaminopyridine
TLC	thin-layer chromatography
DCC	N,N′-dicyclohexylcarbodiimide
HPLC	High-performance liquid chromatography
NMR	Nuclear Magnetic Resonance
