# Supplementary material for: Phospholipid–Prednisolone Conjugates for Targeted IBD Therapy: Structural Design, Synthetic Approaches, Optimization, and Computational Evaluation
Source: Molecules. 2026 Aug 31;31(17):3057. doi: 10.3390/molecules31173057 (PMC13567021; doi:10.3390/molecules31173057)

Supporting information

Phospholipid–Prednisolone Conjugates for Targeted IBD Therapy: Structural Design, Synthetic Approaches, Optimization, and Computational Evaluation

Sapir Ifrah [1], Adi Jabarin [1], Ludmila Yarmolinsky [1], Keren Cohen-Hagai [2], Mordechai Deutsch [2], Arik Dahan [1,*] and Shimon Ben-Shabat [1,*]

[1] Department of Clinical Pharmacology, School of Pharmacy, Faculty of Health Sciences, Ben-Gurion Univer sity of the Negev, Beer-Sheva 8410501, Israel
[2] Department of Nephrology and Hypertension, Meir Medical Center, Kfar Saba 4428164, Israel
* Correspondence: arikd@bgu.ac.il (A.D.); sbs@bgu.ac.il (S.B.-S.)

General procedure for acylation of LPC (A):

4-dimethylaminopyridine (DMAP, 2 mmol, 2.0 equiv) was added to a neat solution of LPC 1 (1 mmol, 1.0 equiv) in the corresponding acid chloride 2a-d (1 mmol, 1.0 equiv) and stirred in dichloromethane (300 mL) at room temperature overnight. The reaction was monitored by TLC. After consumption of the starting material, the mixture was purified using a methanol/water cartridge to afford the desired compounds 3a-d.

LPCA-C4 (LPC-4- Butyric acid) (3a): The title compound was prepared using procedure (A) with LPC **A** (500 mg, 1 mmol), DMAP (244 mg, 2 mmol) and acid chloride **2a** (183 mg, 1 mmol) at RT overnight. Purification by cartridge using 10% methanol/water afforded product 3a as a white solid (330 mg, 48.3%).^{1}H NMR (500 MHz, $CDCl_3$) 5.29 (m, 2H), 4.45-3.70 (m, 8H), 3.26 (s, 9H), 3.19 (s, 11H), 2.4-2.26 (s, 5H), 1.59-1.55 (m, 5H), 1.34-1.17(m, 15H), 0.86 (s, 2H).^{13}C NMR (500 MHz, $CDCl_3$) δ 177.75, 158.00, 141.21, 107.8, 55.2, 54.46, 41.04,35.52, 35.04, 30.66, 30.72, 30.45, 30.34, 30.22, 28.21, 28.17, 25.85, 25.65,15.12. HRMS (ESI) calcd for $C_{30}H_{58}NO_{10}P$ [M+H]+623.38, found 624.38.

LPCA-C4

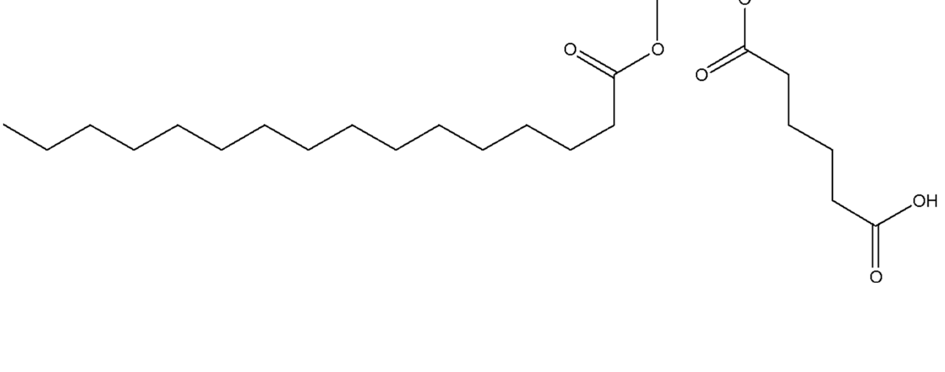

LPCA-C6 (LPC-6- hexanoic acid) **(3b):** The title compound was prepared using procedure (A) with LPC **A** (500 mg, 1 mmol), DMAP (244 mg, 2 mmol) and acid chloride **2b** (211 mg, 1 mmol) at RT overnight. Purification by cartridge using 10% methanol/water afforded product 3b as a white solid (300 mg, 42%).^{1}H NMR (500 MHz, $CDCl_3$) 5.24 (s, 5H), 4.37-3.78 (m,6H) , 3.34 (d, J =5.5, 8H) , 3.18 (s, 12H) ,2.18 (d, J = 10, 6H), 1.49 (d, J = 3, 6H), 1.24-1.13 (m,17H), 0.79 (t, J = 7,5,1, 1H). ^{13}C NMR (500 MHz, $CDCl_3$) δ 177.14, 158.22, 140.26, 131.16, 130.96, 128.96, 128.81, 107.88, 67.10, 60.55, 55.29, 54.52, 41.18, 35.33, 35.26, 34.92, 34.96, 32.83, 32.42, 30.61 ,30.57 ,30.45 ,30.26 ,30.15, 30.07, 30.00, 29.48, 29.25, 29.10 ,28.12 ,26.54 ,25.77 ,25.65 ,

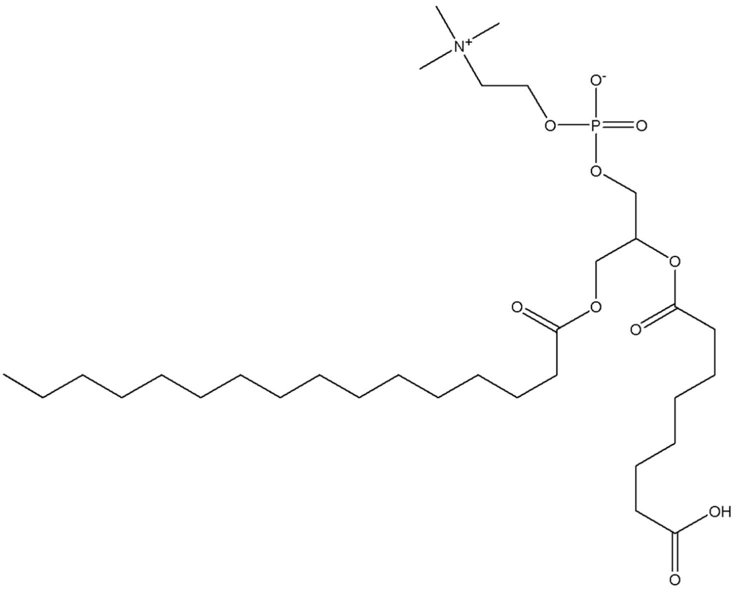

25.55, 25.46, 23.61, 23.49, 15.07, 15.03. HRMS (ESI) calcd for $C_{32}H_{62}NO_{10}P$ [M+H]+651.41, found 652.41.

LPCA-C8 (LPC-8- oxooctanoic acid) **(3c):** The title compound was prepared using procedure (A) with LPC **A** (500 mg, 1 mmol), DMAP (244 mg, 2 mmol) and acid chloride **2c** (239 mg, 1 mmol) at RT overnight. Purification by cartridge using 10% methanol/water afforded product 3c as a white solid (400 mg, 53.98%).^{1}H NMR (500 MHz, $CDCl_3$) 5.29 (t, J = 1.5Hz, 3H), 4.11-3.61(m, 4H), 3.34 (d, J = 5Hz, 6H), 3.23 (t, J = 1.5Hz, 12H), 2.30-2.25 (m, 6H), 2.08-1.93(m, 2H), 1.58-1.52 (m, 6H), 1.23 (d, J = 4.5Hz, 24H), 0.85 (s, 2H). ^{13}C NMR (500 MHz, $CDCl_3$) δ 178.05, 178.01, 158.32, 140.18, 131.22, 131.03, 129.01, 128.87, 107.78, 55.40, 54.46, 41.21, 35.17, 35.02,

30.68 ,30.47 ,30.31 ,30.17 ,30.10, 29.80, 29.75, 29.59, 29.51, 28.18 ,26.6 ,25.84, 25.74 ,25.62 , 23.66, 23.54, 15.08. HRMS (ESI) calcd for $C_{34}H_{66}NO_{10}P$ [M+H]+679.44, found 680.44.

LPCA-C8

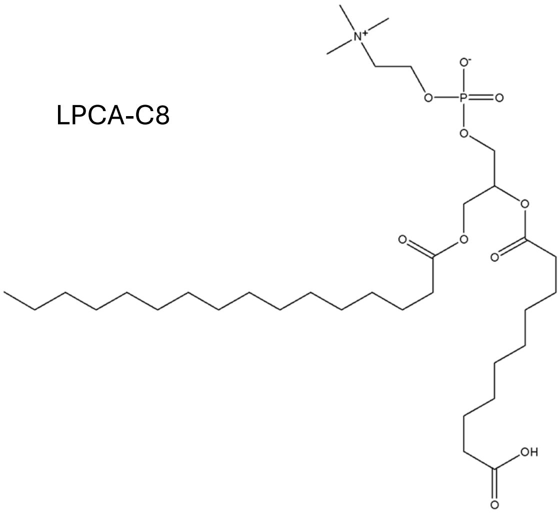

LPCA-C10 (LPC-10- oxodecanoic acid) **(3d):** The title compound was prepared using procedure (A) with LPC **A** (500 mg, 1 mmol), DMAP (244 mg, 2 mmol) and acid chloride **2d** (267 mg, 1 mmol) at RT overnight. Purification by cartridge using 10% methanol/water afforded product 3d as a white solid (360 mg, 47%).^{1}H NMR (500 MHz, $CDCl_3$) 5.29 (s, 1H), 4.15-3.87(m, 6H), 3.40 (s, 8H),3.22 (s, 15H), 2.27 (t, J = 7Hz, 5H), 1.58 (t, J = 6.7Hz, 6H),1.32-1.21 (m, 26H), 0.94-0.85 (m, 2H). ^{13}C NMR (500 MHz, $CDCl_3$) δ 178.04, 158.12, 140.89, 107.70, 67.21, 67.25, 60.63, 55.48, 41.10, 35.65, 35.13, 35.02, 30.69 ,30.65 ,30.52, 30.34 ,30.25 ,30.21, 30.16, 29.98, 29.84, 29.69, 28.19 ,25.96 ,25.88, 15.12, 15.08. HRMS (ESI) calcd for $C_{36}H_{70}NO_{10}P$ [M+H]+708.47, found 707.47.

LPCA-C10

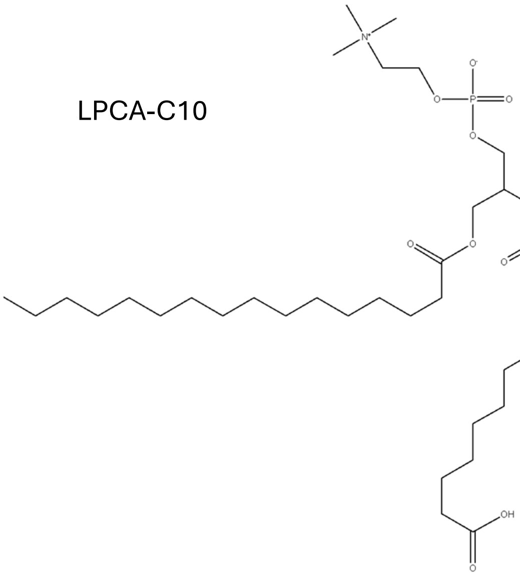

Supplement: Supplementary file 1 [file molecules-31-03057-s001.zip › molecules-4381850-supplementary.pdf]
